# Do Flipped Learning and Adaptive Instruction Improve Student Learning Outcome? A Case Study of a Computer Programming Course in Taiwan

**DOI:** 10.3389/fpsyg.2021.768183

**Published:** 2022-01-14

**Authors:** Hong-Ren Chen, Wen-Chiao Hsu

**Affiliations:** ^1^Department of Digital Content and Technology, National Taichung University of Education, Taichung, Taiwan; ^2^Department of Information Management, National Taichung University of Science and Technology, Taichung, Taiwan

**Keywords:** flipped learning, adaptive instruction, computer programming, learning outcome, teaching strategies

## Abstract

Flipped learning could improve the learning effectiveness of students. However, some studies have pointed out the limitations related to flipped classrooms because the content of the flipped course does not vary according to the needs of the students. On the other hand, adaptive teaching, which customizes the learning mode according to the individual needs of students, can make up for some of the shortcomings of flipped teaching. This study combines adaptive teaching with flipped teaching and applies it to face-to-face classroom activities. The purpose of this research is to explore whether the implementation of flipping and adaptive learning in a computer programming course can improve the learning effectiveness of students. The experimental subjects of this study are the sophomore students in the Department of Information Management. The flipped classroom with adaptive instruction has been realized in the limited course time. This study uses questionnaires to collect pre- and post-test data on the “learning motivation” of students. The learning effectiveness was evaluated based on the students' previous programming course (C language) and the semester scores of this course. Research results show that the post-test “learning motivation” has improved overall compared with the pre-test, and the learning effect is significant. The results of this research not only prove the effectiveness of modern teaching theories in programming courses but also lay the foundation for future teaching design.

## Introduction

For a world where technology is omnipresent, more education systems recognize the importance of computer science. Computer science education is expanding around the globe. For example, improving the coding and programming ability of students is an important goal of countries (Mechaber, 2014). Different from popular technology education and basic knowledge of programming teaching, the goal of programming courses in information technology departments of colleges and universities is to train professional programming talents. Programming is an important basic skill for computer science students. It is one of the most difficult subjects to learn because it involves skills, such as deducing algorithms, understanding syntax and semantics, and coding programs (Daly, 1999; Jenkins, 2002). There are certain learning thresholds for learning programming languages. Some students often encounter bottlenecks in the learning process and lose interest in learning (Kadar et al., 2021). Rahma et al. (2012) explored the major problems affecting the performance of students in basic programming. In addition to the characteristics of the programming discipline itself, it also includes the factors of students and teachers. The learning style of students varies from student to student and is one of the factors that affect learning. Learning interest and motivation are also key factors, which are related to the way the teachers conduct the class. Sarpong et al. (2013) found that the most effective teaching methods for teaching programming courses are as follows: laboratory practice, projects, lectures, seminars, and tutorials, and problem-based teaching. The most suitable strategies for teaching programming courses are problem-based teaching and pair/group programming. To improve the learning outcomes of computer programming courses, Sarpong et al. (2013) recommended that teachers use more than one teaching method or strategy in the class.

How to improve the learning effectiveness of students and reduce the frustration of learning programming has always been a goal that teachers have achieved. In recent years, there are many approaches to improve the learning outcome of the student through active learning methods. One of the methods is the flipped classroom method. In addition, there are many studies indicating that flipped classrooms provide many positive educational results (Tune et al., 2013; O'Flaherty and Philips, 2015; Seery, 2015). Zhang et al. (2016) demonstrated that most students agree that flipped learning is a useful teaching method, where it was considered to be particularly useful for hands-on learning (such as programming). However, some studies have pointed out the challenges and difficulties related to flipped classrooms (Lo et al., 2018; Cheng et al., 2019). Mainly because there are differences among students in a class. The content of the flipped course does not emphasize to vary according to the needs of the students.

Based on the above discussion, it is known that programming courses are suitable for flipped learning, and a mixed teaching method is a good choice. In many teaching strategies, adaptive teaching emphasizes that teaching methods and skills should meet the needs of different students, which can make up for some of the shortcomings of flipped teaching. Because it can customize the learning mode according to the individual needs of students, the value of adaptive learning is generally affirmed in higher education. Although neither the flipped classroom nor the adaptive teaching is a new teaching theory, there are few related studies combining the two in the literature. A search was conducted on Web of Science (WoS) for English papers from 1988 to July 2021 by the search term of “TS = (flip ^*^ AND (learn ^*^ OR classroom)) AND (adaptive AND (learn ^*^ OR instruction))”. There are 48 search results. Only 17 left after removing the completely irrelevant results. Six of these papers mainly study flipped learning (Louhab et al., 2018; Rodriguez et al., 2018; Lamia et al., 2019; van Leeuwen, 2019; Janson et al., 2020; Ranellucci et al., 2021), and one is about adaptive learning (Alwadei et al., 2020). Among the 10 papers on these two topics (flipped and adaptive), three are reviews or discourse analysis papers (Chi et al., 2018; Narang et al., 2018; Smale-Jacobse et al., 2019), and seven focus on the topic of flipped teaching combined with adaptive e-learning platforms (Fang et al., 2019; Kaw et al., 2019; Clark and Kaw, 2020; Louhab et al., 2020; Mojtahedi et al., 2020; Hsieh et al., 2021; Liu et al., 2021). There is almost no research in the literature on the combination of flipped learning and adaptive teaching in the classroom.

The motivation of this research is to explore whether the implementation of hybrid methods in computer programming courses, that is, flipped learning and adaptive teaching, can improve the learning efficiency of students. The main objectives of this research are as follows:

(1) Discuss the impact of combining flipped learning and adaptive teaching on the academic performance of students.
(2) Discuss the influence of combining flipped learning and adaptive teaching on the learning motivation of students.
(3) Explore perceptions of students about the class in terms of content, communication, performances, and interests.

The experimental subjects of this study are the sophomore students in the Department of Information Management. Students preview the teaching materials provided by teachers before class. In the classroom, the teacher taught the main points of the lectures. Then, conducting problem-solving or higher-level critical thinking activities individually or in groups. Before the midterm, students mainly conduct problem-solving training. After the midterm, students were divided into two groups based on the midterm results. Flip teaching was still ongoing, and the class frees up class time for adaptive teaching activities. Students with a score lower than or equal to 70 in the midterm exam are in the first group, and students with a score higher than 70 are in the second group. The teaching focuses for the first group were on strengthening the training of basic programming skills. The second group is to solve more complex problems in groups. This research uses the questionnaire survey method to collect students' pre- and post-test data of “learning motivation” and post-test data of perceptions of students about the classroom. The learning performance was evaluated based on the students' previous programming course (C language) and the semester scores of this course. Research results show that the post-test “learning motivation” has improved overall compared with the pre-test. Students are highly satisfied with this course and the learning effect is also significant.

The main contribution of this study can be summarized as follows:

(1) This research proposes a hybrid teaching method to improve the shortcomings of flipped learning that lacks consideration of individual differences through adaptive instruction.
(2) As far as we know, no research work has considered a hybrid method of flipped learning and adaptive instruction applied to the face-to-face classroom activities in computer programming courses. This case is supposed to be the first flipped classroom that realizes adaptive teaching in a limited course time.
(3) The results of this research confirm that the hybrid teaching method helped to improve the learning motivation and academic performance of students. In addition, students are highly satisfied with the way the course was conducted.
(4) The results of this research have proved the effectiveness of modern teaching theories in programming courses. The research results will be used to improve the curriculum to ensure the realization of the curriculum effect and improve students' academic performance and motivation to learn programming.

## Literature Review

### Flipped Learning

The flipped classroom is a learning model which reversed or flipped classroom activities and homework (Jensen et al., 2018). In traditional teaching, instructors teach knowledge in the classroom, and students do their homework after class. In the flipped model, instructors have students learning or doing homework first, followed by discussing during class time and putting some ideas into practice (Kong, 2014; Sever, 2014; Çevikba, and Argün, 2017; Yavuz and Ozdemir, 2019). The flipped classroom provides some advantages. Class time management has become more effective, saving learners time in participating and collaborative activities (Baker, 2000; Cole and Kritzer, 2009; Fulton, 2012; Milman, 2012; Halili and Zainuddin, 2015). The interaction between learners and teachers has improved (Lage et al., 2000; Bergmann and Sams, 2012; Roehl et al., 2013; Arnold-Garza, 2014). Moreover, it helps teachers to immediately monitor learner performance and tutor learners with difficulties (Lage et al., 2000; Fulton, 2012; Millard, 2012).

Zhang et al. (2016) believe that flipped learning is particularly useful for programming courses. Many works proved their effectiveness. Mok's study (Mok, 2014) evaluated the effects of flipped classrooms in programming courses based on the perspectives of students. The study concluded that this method is effective for learning programming, the participation rate of students in the learning process is higher with higher satisfaction of students. Souza and Rodrigues (2015) compared the experimental research of flipped teaching and traditional teaching in C programming courses. According to the research results, compared with traditional teaching methods, the flipped classroom significantly improves the programming self-efficacy and academic success rate of students. Chiang (2017) analyzed the impact of flipped learning on the problem-solving ability of students in Java programming learning. The experiment concluded that the problem-solving strategies of students are more effective than their previous problem-solving attitudes. Özyurt and Özyurt (2018) proposed an Adapted flipped classroom approach (AFCA), which analyzes the effects of the use of AFCA on the programming success, attitudes, and self-efficacy of the students in learning programming. The study results show that employing AFCA to teach programming yields positive effects in terms of the programming success and self-efficacy of students. There was no significant change in their attitude scores following the implementation.

Although flipped classrooms have brought some positive effects on learning, some studies have raised some challenges. For example, Milman (2012) mentioned that if learners play computer games while watching instructional videos, learners will be distracted by them and the learning process will be hindered. In addition, Moffett and Mill (2014) believed that if learners are unwilling to experience online learning, the learning effect will not improve. Moore and Chung (2015) mentioned that every learner has a different learning style, and flipped learning may not be able to respond to the needs and preferences of learners. Jawawi et al. (2015) pointed out that learners may have limited access to the tools or resources needed for online learning, which hinders flipped learning (Kissi et al., 2017).

To ensure that flipped learning or flipped classrooms can achieve better results, some studies have begun to adopt hybrid flipped teaching methods. For example, Miras et al. (2021) designed a hybrid teaching method of flipped classroom and peer instruction in an introductory course of programming. Comparing with those obtained in the previous offerings using traditional teaching, the results showed that the new methodology was used, the dropout rate and fail share decreased significantly and the academic results have improved. This study adopted a hybrid strategy. To solve the inadequate consideration of individual differences in flipped learning, adaptive teaching was combined in the classroom to reinforce it.

### Adaptive Instruction

Adaptive instruction means creating a learning environment and finding instructional approaches and techniques that conform to meet individual needs of students (Inan and Grant, 2008). It can be adaptive teaching or adaptive learning (Matei and Gogu, 2017). In the teaching process, in accordance with the abilities, status, interests, and needs of learners, appropriate responses and adjustments are made to improve the learning effect and achieve the expected teaching goals. Parsons et al. (2018) identified that early literature focused on the decision-making of teachers. Between 1995 and 2004, literature often referred to scaffolding and teacher reflection. Until 2008, adaptive teaching started being mentioned in literature. There are some major issues usually discussed in terms of adaptive teaching, such as how to evaluate adaptive teaching, how to judge the degree of success of adaptive teaching for individual students, what are the relations between adaptive teaching and professional competence of teachers, and which methodological approaches are useful for investigating outcomes of adaptive teaching (Hardy et al., 2019). In addition, smart devices and intelligent technologies were applied to effectively promote the development of personalized learning and adaptive learning. Peng et al. (2019) introduced a teaching method enabled by a smart learning environment and deeply analyzed personalized learning and adaptive learning. Four aspects, which are learner profiles, competency-based progression, personal learning, and flexible learning environments, constructed personalized adaptive learning. Peng et al. explored a form of learning profiles model and a generative path recommendation pattern of personal learning.

There is almost no literature discussing the implementation of adaptive teaching in the classroom of programming teaching. It may be because it is difficult to implement adaptive teaching alone in a limited time class. When discussing “adaptive” and “computer programming learning” in the literature, most of them talk about an adaptive web-based system or an adaptive e-learning platform for learning programming (Chatzopoulou and Economides, 2010; Troussas et al., 2021). Although we can find relevant papers by searching with “flipped learning” and “adaptability” as keywords, they also talk about the use of adaptive platforms to assist students in studying off class (Fang et al., 2019; Kaw et al., 2019; Clark and Kaw, 2020; Louhab et al., 2020; Mojtahedi et al., 2020; Hsieh et al., 2021; Liu et al., 2021).

### Learning Effectiveness

Learning effectiveness is an index used to judge the learning achievement of learners. After learners participate in learning, their learning status can be measured by evaluating changes or differences in the performance of these indicators. Based on the results of indicators, learners could improve learning methods and teachers could improve teaching methods (Guay et al., 2008). Kirkpatrick and Kirkpatrick (2006) proposed a four-level training evaluations model, which includes four items: reaction, learning, behavior, and results. The reaction aspect refers to the degree to which learners like the course. The learning aspect refers to whether learners gain knowledge accumulation or mental growth. The behavior aspect refers to whether learners have learned to change their behavior. Result aspect refers to the degree to which learners can apply the content of learning. The learning effects discussed in this research include the perceptions of students about the class (reaction aspect), academic performance (learning aspect), and learning motivation (learning aspect).

Students' perception of the course is related to their satisfaction with the course. Student satisfaction can be defined as the attitude of students on the course learning experience, teacher's teaching quality, and teaching materials (Gao et al., 2021). Many pieces of literature use the opinions of students as the basis for measuring the effectiveness of the course implementation (Mok, 2014; Choe et al., 2019; Gao et al., 2021). Academic performance is the most direct result of what students learn. Additionally, it is one of the most used indicators to measure learning outcomes (Hwang et al., 2013; Cheng et al., 2019; Choe et al., 2019; Miras et al., 2021). The success of learning depends on the enthusiasm of the learner and motivation drives learners to achieve learning goals. Therefore, the learning motivation of learners is probably the important key to affecting learning effectiveness (Filgona et al., 2020). Hadre et al. (2007) believe that motivation is one of the most powerful factors that determine the success or failure of students in school. Stimulating the enthusiasm of students for learning in school and motivating students to succeed in school is an important issue, and it is also one of the biggest challenges facing education (Filgona et al., 2020). There are many studies in the literature on how to enhance the learning motivation of students (Jensen et al., 2018; Awidi and Paynter, 2019; Fang et al., 2019; Hardy et al., 2019; Ling et al., 2021; Miras et al., 2021), which shows the degree of importance it is paid to.

## Methodology

### Background Circumstances for the Classroom Design

Our flipped classroom study involved 52 sophomores of the Information Management Department in a Java programming course. Participating students have taken a programming course (C language) in the freshman year, so they have the basic concepts of programming. To understand the impact of the design of this experimental course on the learning performance of students, the grades of the previous programming course were used as the pre-test data and the semester grades of this experimental course were used as the post-test data of “learning performance.”

The first step of the teaching process was drawing up the topics and goals of the weekly courses. The online teaching material would be uploaded for students to study in advance. The beginning of the 3-h classes is a simple test for the preview result of students. The main purpose is to urge students to read the teaching material before the classes. After the test, the teacher will reinforce the important content. Some classroom activities will be arranged afterward, which were different before and after the midterm exam. Before the midterm exams, students mainly strengthen their programming foundation and problem-solving skills. Therefore, the activities were mainly homework exercises or small competitions. After the midterm exam, the students were divided into two groups based on their midterm exam results. Students with a score of 70 or more are classified into the “Achievement challenge group,” and the others are classified into the “ability improvement group.” The learning content before the midterm exam focuses on understanding Java and rarely involves more complicated logic problems. Based on previous teaching experience, students with mid-term grades above 70 will be able to deal with complex problems. Students with a mid-term score of 70 or less still need to strengthen their basic programming skills to pass the exam at the end of the term. Therefore, 70 points are used as the grouping limit. Then, the course adopted the concept of adaptive teaching and performed different classroom activities. Students in the “Achievement challenge group” challenged more difficult learning tasks in groups. At the same time, students in the “Ability improvement group” practiced more basic topics and were guided by teachers. Before the end of the course, the “Achievement challenge group” shared the results of the challenge and the difficulties encountered in the process. After discussing with the students, the teacher made a review and summary of this week's course and previewed the course of next week.

### Research Tools

The research tools in this study included a pair of pre- and post-grades, a pair of pre- and post-test questionnaires for measuring the learning motivation of students, and a post-test questionnaire for perceptions of students on the course.

The data of pre-test of learning performance of students were collected from the semester grade of programming course (C language) they had taken in the freshman year. The semester grade of this experimental course was the post-test data of learning performance. As the two control variables (course time, teaching materials, measuring methods, and teachers) were not the same, in addition to the original grade for verification, the pre- and post-test grades were normalized by the Min-Max. Min-max normalization is one of the most common ways to normalize data. The minimum value and the maximum value of grades get transformed into a 0 and a 1, respectively. Therefore, every grade would be transformed into a decimal between 0 and 1. To make it easier to compare with the original grade, the normalized value was multiplied by 100. The formula is (x − min)/(max − min) ^*^ 100. Then, a paired *t*-test was performed to detect the impact of this course on the academic performance of students.

The questionnaire of learning motivation was from the paper published by Hwang et al. (2013), which was modified according to the measurement method developed by Hwang and Chang (2011). There was a total of seven questions with a six-point rating scheme (6 = “strongly agree” and 1 = “strongly disagree”). The items in the questionnaire are listed below. The content of this questionnaire contains values (e.g., item 1, 3, and 7), expectations (e.g., item 2 and, 6), and emotions (e.g., item 4 and 5) (Eccles, 1983) to evaluate the mental thinking of students about learning Java. Cronbach's alpha value was 0.79. At the beginning of the course, the students took the pre-test of learning motivation questionnaire. At the end of the course, the students took the post-test of the learning motivation questionnaire. Compare the differences to understand the impact of curriculum activities on the learning motivation of students.

1. I think learning Java programming is interesting and valuable.
2. I would like to learn more and observe more in the Java programming course.
3. It is worth learning those things about Java programming.
4. It is important for me to learn the Java programming course well.
5. It is important to know the Java programming knowledge related to our living environment.
6. I will actively search for more information and learn about Java programming.
7. It is important for everyone to take the Java programming course.

To understand the views of students on the design of this course, students took a questionnaire at the end of the course. We used the “questionnaire of students' perceptions of the online community-based flipped classroom” introduced by Lin and Hwang (2018). The questionnaire evaluates the students' perceptions of flipped learning by a five-point rating scheme (5 = “strongly agree” and 1 = “strongly disagree”) with 14 items. To be consistent with the rating scheme of the learning motivation questionnaire, a six-point rating scheme was used during the actual test. These 14 items were divided into four dimensions. Items 1–5 are for content dimension, which test whether the student thinks the content of the course is helpful. Items 6–8 are for communication dimension, which test whether the student thinks that the curriculum design is helpful for communication with teachers and peers. Items 9–11 are for performance dimension, which measure whether the student thinks that the course contributes to their personal performance. Items 12–14 are for interest dimension, which measure whether the student thinks the class is enjoyable. Cronbach's α values of the individual dimensions were 0.85, 0.79, 0.78, and 0.87, respectively, showing acceptable reliability in internal consistency. The items of each dimension are as follows:

#### Content

1. The designed classroom offers me the opportunity to review the lectures as many times as I need.
2. The designed classroom offers me access to the online course tools and materials.
3. The designed classroom helps me to use various e-learning resources.
4. The designed classroom helps me to enrich my learning experience.
5. The designed classroom helps me to connect theory with practice in real life.

#### Communication

6. The designed classroom helps me to effectively cooperate with my classmates and colleagues.
7. The designed classroom facilitates more communication between me and my teacher.
8. The designed classroom helps me to effectively participate in the learning activities.

#### Performances

9. The designed classroom enables me to manage my own learning activities.
10. The designed classroom helps me to develop my problem-solving skills.
11. The designed classroom facilitates more communication between me and my classmates.

#### Interests

12. The designed classroom is a very enjoyable approach.
13. I prefer the designed classroom over the traditional lectures.
14. The designed classroom facilitates my personalized learning.

## Results

### Analysis of Learning Performance

In this study, the semester grade of the previous programming course taken by the students was the pre-test value of the “learning performance,” the semester grade of the experimental course was the post-test value. The original grades were normalized by the Min–Max. Both grades performed the paired sample *t*-test.

As shown in Table 1, the mean values and SDs of the original grades were 72.60 and 10.22 for the pre-test, and 77.93 and 13.93 for the post-test. The *t*-test result (*t* = 3.68, *p* < 0.05) shows that there was a significant difference between the two tests. The result *t*-test of normalized grade (*t* = 4.53, *p* < 0.05) yielded the same result. The average score increased from 50.27 to 64.69, and the SD decreased from 29.19 to 26.48, indicating that students generally performed better after the course, and the distribution of grades was more concentrated.

**Table 1:** Paired *t*-test result of the grades.

**Group**	***N***		**Test**	**Mean**	***S.D*.**	***t***	***P***
All students	52	Original grade	Pre-test	72.60	10.22	3.68	0.000^*^
			Post-test	77.93	13.93		
		Normalized grade	Pre-test	50.27	29.19	4.53	0.000^*^
			Post-test	64.69	26.48		
Achievement challenge	30	Original grade	Pre-test	77.17	9.71	5.83	0.000^*^
			Post-test	87.68	7.56		
		Normalized grade	Pre-test	63.33	27.74	4.17	0.000^*^
			Post-test	83.24	14.38		
Ability improvement	22	Original grade	Pre-test	66.36	7.27	−1.29	0.105
			Post-test	64.62	8.40		
		Normalized grade	Pre-test	32.46	20.76	2.13	0.022^*^
			Post-test	39.40	16.00		

* *p <0.05. The bold value indicates that the experiment is significant (p <0.05)*.

After the midterm exam, the participating students were divided into two groups according to their grades in the midterm exam. There are 30 students in the “Achievement challenge” group with the grade higher than or equal to 70. There are 22 students in the “Abilities Improvement” group with the grade lower than 70. After the midterm exam, adaptive instruction was performed. To better understand the two groups of the learning performances of students, the two groups, respectively, performed *t*-tests of their semester grades. The results are shown in Table 1.

In the “Achievement challenge” group, the means and SDs of the original grade were 77.17 and 9.71 for the pre-test, and 87.68 and 7.56 for the post-test. The means and SDs of the normalized grade were 63.66 and 27.74 for the pre-test, and 83.24 and 14.38 for the post-test. Both *t*-test results of before standardization (*t* = 5.83, *p* < 0.05) or after standardization (*t* = 4.17, *p* < 0.05) showed that students have made significant progress in academic performance after the course. In the “Ability improvement” group, the means and SDs of the original grade were 66.36 and 27.74 for the pre-test, and 64.62 and 8.40 for the post-test. Although the average grade of the original has dropped, it has not reached a significant level (*t* = −1.29, *p* > 0.05). However, the *t*-test result of normalized grade (*t* = 2.13, *p* < 0.05) showed significantly better learning performance after the course.

The above analysis shows that the design of this course in terms of adaptive teaching has a considerable degree of help for students with different levels. It has a significant improvement of the “achievement challenge” group. However, for the “capacity improvement” group, there was a relatively limited degree of improvement.

### Analysis of Learning Motivation

Table 2 shows the *t*-test results of the pre- and post-test ratings of the learning motivation questionnaire for all students and the two groups. The means of the questionnaire ratings have increased regardless of whether statistics were conducted in all or groups, which show that the learning motivation of students has improved. The *t*-test results showed significant difference between pre- and post-test ratings for all students and both groups, where *t* = 3.75 (*p* < 0.05) and *t* = 3.70 (*p* < 0.05), and *t* = 1.74 (*p* < 0.05), respectively. The results showed that the introduction of flipped learning and adaptive teaching in the course has a positive and significant effect on the improvement of the learning motivation of students.

**Table 2:** Paired *t*-test result of the learning motivation.

**Group**	***N***	**test**	**Mean**	**S.D**.	***t***	***P***
All students	52	Pre-test	4.42	0.68	3.75	0.000^*^
		Post-test	4.72	0.68		
Achievement challenge	30	Pre-test	4.70	0.59	3.70	0.000^*^
		Post-test	5.05	0.61		
Ability improvement	22	Pre-test	4.04	0.60	1.74	0.048^*^
		Post-test	4.28	0.54		

* *p <0.05. The bold value indicates that the experiment is significant (p <0.05)*.

### Analysis of Students' Perceptions

**Figure 1:**
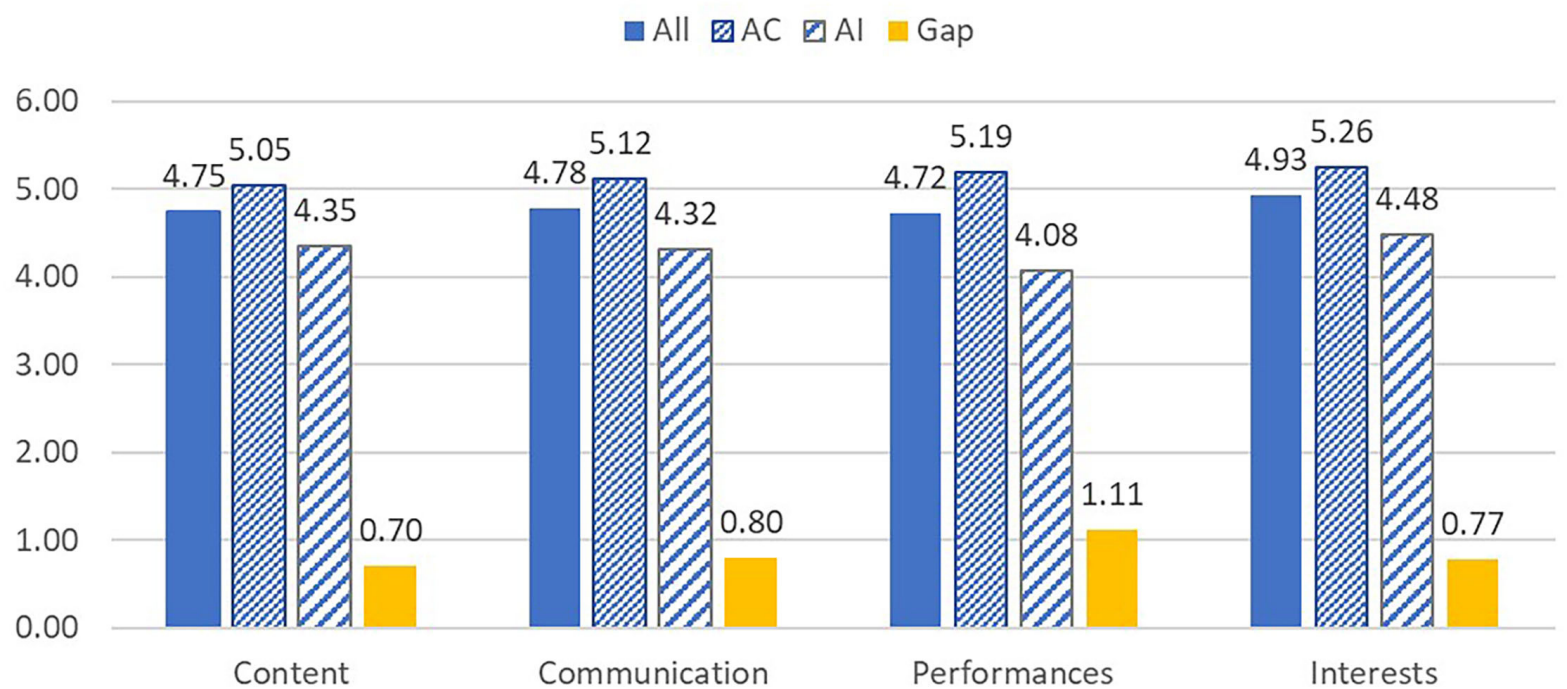
Histogram of the average scores of four dimensions of students' perceptions. All means all students. AC means the achievement of the challenge group. AI means the ability of improvement group. The gap means the difference of AC to AI.

As it was a new experience for the students to learn under the flipped learning combined with the adaptive instruction, it is interesting to know the views of the students about the class. There are 14 items in the questionnaire of perceptions of students about the course, which are divided into four dimensions: content, communication, performances, and interest. This study adopted the six-point rating scheme. The questionnaire was administered at the end of the course. The average rating of four dimensions of perceptions of students about the course is shown in Figure 1. Overall, students' perception of curriculum design is positive. Among the four dimensions, the average scores are 4.75, 4.78, 4.72, and 4.93, respectively. Students were most satisfied with the “interest” aspects of the course. The average ratings for each aspect of the achievement challenge group are all greater than the ability improvement group. The dimension with the least difference is content, and the dimension with the greatest difference is performance. This result is reasonable because the teaching materials provided in the course were the same. Meanwhile, the achievement challenge group has more discussion and problem-solving training.

## Discussion and Conclusion

The flipped classroom method has become a popular teaching method in many educational institutions around the world. Comparing learning outcomes with traditional teaching, most previous reviews indicate that the flipped classroom approach can improve student performance. However, flipped learning is not effective for all students (Gundlach et al., 2015). The review paper by Lo and Hew (2017) suggested that the necessary help and guidance should be given based on the abilities of students. To improve the effect of flipped learning, this study designed a teaching method that incorporates adaptive teaching into flipped classrooms. Students with better programming skills (achievement challenge group) have a better understanding of the content of the course increasing the pair/group programming and problem-solving challenges. Students with weaker programming skills will strengthen their logic and skills of solving problems, increasing the amount of practice and opportunities for interaction with the teacher. The research questions of this study are to explore whether the hybrid teaching method can improve the performance and learning motivation of students and collect students' perceptions of the curriculum design.

In terms of learning performance, according to the data analysis results of this research, the hybrid teaching method of flipped learning and adaptive instruction was effective for improving the academic performance of students. Not only effective for the achievement challenge group but also effective for the ability improvement group. In terms of grouping, the performance improvement of the achievement challenge group is greater than that of the ability improvement group. It means that the teaching method of this study has a higher impact on students with better programming ability than students with weaker programming ability.

According to the previous studies, Hsieh et al. (2021), Awidi and Paynter (2019), and Lamia et al. (2019) have indicated that the flipped classroom enhances the learning motivation of learners. In our flipped classroom, students participate in different classroom activities according to their abilities. The overall learning motivation has been significantly improved, whether it is analyzed by all students or by grouping. However, we found that the improvement of learning motivation in the ability improvement group was lower than that of the achievement challenge group. The difference may be due to adaptive teaching adopting different activities for the two groups, or it may be because it is relatively difficult to improve the motivation of students with weaker programming abilities to learn programming.

In terms of students' cognition of the classroom, students generally agree with classroom teaching in terms of content, communication, performance, and interest. Past studies have also shown that the satisfaction of students with flipped classrooms is high (Özyurt and Özyurt, 2018). The questionnaire used in this study comes from Lin and Hwang (2018), which showed the ranking of average satisfaction from high to low in interest, content, communication, and performance. In this study, for all students, the average satisfaction ranking by descending is interest, communication, content, and performance. Both studies show that students have the highest satisfaction in terms of interest. Due to the implementation of adaptive teaching in this study, the two groups of students have different degrees of satisfaction in each dimension. Excluding the interest dimension, the achievement challenge group was more satisfied with the implementation and communication than the content. On the other hand, the ability improvement group was more satisfied with the content and communication than the implementation. The difference is mainly caused by adaptive teaching that involves different groups of students in different activities.

In summary, this study adopted a mixed teaching method of flipping and adaptability, which significantly improves the academic performance and learning motivation of students. In addition, students are highly satisfied with the course. Our research successfully provided an effective case study of the hybrid teaching method. There are some limitations to this study. First, it is a pre-test-post-test set of experimental case study. Therefore, it cannot be compared with the effect of traditional teaching courses. Multi-group analysis will be a topic worth discussing in the future. Second, it only contains quantitative data analysis. In the future, it is possible to design mixed studies that use both qualitative and quantitative methods. Third, the small number of samples in the study and the implementation of the experiment only in a Java course is another limitation. In the future, it will be interesting to collect the learning status of more students and get more general analysis results.

## Data Availability Statement

The raw data supporting the conclusions of this article will be made available by the authors, without undue reservation.

## Ethics Statement

The studies involving human participants were reviewed and approved by Teaching Practice Research Program, Ministry of Education, Republic of China (Taiwan). The patients/participants provided their written informed consent to participate in this study.

## Author Contributions

H-RC contributed to the research topic and the methodology. W-CH contributed to the research model and the experimental design and results. H-RC and W-CH contributed to the statistical analysis and the discussion. All authors contributed to the article and approved the submitted version.

## Funding

This work was supported in part by the Ministry of Education of the Republic of China (Contract No. PEE107036), and in part by the National Taichung University of Science and Technology (Contract No. NURTURE110-04).

## Conflict of Interest

The authors declare that the research was conducted in the absence of any commercial or financial relationships that could be construed as a potential conflict of interest.

## Publisher's Note

All claims expressed in this article are solely those of the authors and do not necessarily represent those of their affiliated organizations, or those of the publisher, the editors and the reviewers. Any product that may be evaluated in this article, or claim that may be made by its manufacturer, is not guaranteed or endorsed by the publisher.
